# Evidence-Based, Patient-Centered Treatment of Erythema Migrans in the United States

**DOI:** 10.3390/antibiotics10070754

**Published:** 2021-06-22

**Authors:** Elizabeth L. Maloney

**Affiliations:** Partnership for Tick-Borne Diseases Education, P.O. Box 84, Wyoming, MN 55092, USA; ptde.emaloney@gmail.com

**Keywords:** erythema migrans, amoxicillin, cefuroxime, doxycycline, treatment, Lyme disease, antibiotic, patient-centered

## Abstract

Lyme disease, often characterized as a readily treatable infection, can be a debilitating and expensive illness, especially when patients remain symptomatic following therapy for early disease. Identifying and promoting highly effective therapeutic interventions for US patients with erythema migrans (EM) rashes that return them to their pre-infection health status should be a priority. The recently released treatment recommendations by the Infectious Diseases Society of America/American Academy of Neurology/American College of Rheumatology (IDSA/AAN/ACR) for the treatment of US patients fall short of that goal. This paper reviews the US trial evidence regarding EM rashes, discusses the shortcomings of the IDSA/AAN/ACR recommendations in light of that evidence and offers evidence-based, patient-centered strategies for managing patients with erythema migrans lesions.

## 1. Introduction

Lyme disease is an ever-increasing public health threat. A recent estimate from the Centers for Diseases Control and Prevention (CDC) suggests that 476,000 new cases of Lyme disease occur annually in the United States [1]. Although it is often characterized as a readily treatable infection, Lyme disease can be a debilitating and expensive illness, especially when patients present with late manifestations or remain symptomatic following therapy for early disease [2,3,4,5]. In the United States (US), annual direct medical costs, corrected for inflation and increased case numbers, may reach $1.57 billion [1]. Direct medical costs increase significantly when therapy fails to return patients to their pre-Lyme baseline [1]. More importantly, the bulk of Lyme-related costs are due to indirect medical costs, nonmedical costs and lost productivity, and these costs increase with long-standing disease [2]. Thus, the total costs associated with Lyme disease are staggering.

In light of the physical and financial costs, identifying and promoting highly effective therapeutic interventions for US patients with erythema migrans (EM) rashes that return them to their pre-infection health status should be a priority. However, recently released treatment recommendations by the Infectious Diseases Society of America/American Academy of Neurology/American College of Rheumatology (IDSA/AAN/ACR) for the treatment of US patients fall short of that goal [6]. This paper reviews the US trial evidence regarding EM rashes, discusses the shortcomings of the IDSA/AAN/ACR recommendations in light of that evidence and offers evidence-based, patient-centered strategies for managing patients with erythema migrans lesions.

## 2. Background

The IDSA/AAN/ACR issued two recommendations regarding antibiotic therapy for patients with erythema migrans rashes [6]. The first recommendation identified their preferred agents—oral doxycycline, amoxicillin, or cefuroxime axetil. The second specified the duration of therapy: either 10 days of doxycycline or 14 days of either amoxicillin or cefuroxime axetil. If using azithromycin, a second-line agent, the recommended duration is 7 days.

To appreciate the paucity and low quality of the evidence underlying the IDSA/AAN/ACR recommendations for management of EM rashes, one must understand nuances of the infection, the principles of evidence-based medicine and patient-centered care and critical elements of trial design.

Lyme disease results from an infection by one of the many pathogenic species in the *Borrelia burgdorferi sensu lato* complex. In the US, *B. burgdorferi sensu stricto* (Bbss), with rare exceptions, is responsible for all cases of Lyme-related EM rashes [7]. The situation in Europe is more complex. The most prevalent *Bbsl* species include *B. garinii*, *B. afzelii* and Bbss [7]. However, European EM rashes are more commonly due to *B. afzeli* than Bbss [8,9]. Compared to the US EM patients, those with EM rashes due to a *B. afzelii* infection have a milder clinical course [10] and *B. afzelii* is less likely than *B. burgdorferi* to cause disseminated disease [11].

When promptly diagnosed and appropriately treated with antibiotics, early Lyme disease is curable. Untreated and inadequately treated infections can result in disease progression and long-term sequalae [12,13]. The EM rash is the hallmark finding of early disease; other common manifestations include flu-like symptoms such as fever, chills, fatigue, headache, myalgias and arthralgias; neurologic symptoms such as paresthesia have also been reported [14,15,16,17]. EM rashes are not disfiguring, rarely cause pain and resolve without antibiotics.

Patient-centered care, as defined by the National Academy of Medicine (NAM, formerly known as the Institute of Medicine) is “respectful of and responsive to individual patient preferences, needs, and values and ensuring that patient values guide all clinical decisions” [18]. Patient-centered outcomes are outcomes that matter to patients, such as reductions in symptoms or improvements quality of life [19]. Evidence-based medicine is defined as the integration of three elements: best clinical research, clinical expertise and patient values [20]. The relative value of these elements is in tension. When the clinical research evidence is strong, clinical expertise and patient values carry less weight because they would likely align with the research findings. Conversely, when the research evidence is weak, clinical expertise may diverge from the trial findings and the spectrum of patient values may be broad. In such instances, clinical expertise and patient values assume greater importance. Taken together, when possible, the practice of patient-centered, evidence-based medicine should be based on therapies that have demonstrated their effectiveness on patient-centered outcomes in high-quality trials. If high-quality trials are absent, therapeutic options should reflect clinician expertise in conjunction with patient values.

A crucial part of trial design is the selection of appropriate endpoints. Historically, it was common practice for clinical trials to utilize disease-centered outcomes, which are often surrogate outcomes that may or may not reflect actual improvement in a patient’s health [19]. Surrogate outcomes have the advantage of being easily measured. More recently, patient-centered outcomes are being incorporated into trial designs. These types of outcomes are measured using clinically validated tools. With regard to the treatment of EM rashes, given the benign nature of the rash itself, the primary patient-centered therapeutic goals include the restoration of health to its pre-Lyme disease status and the avoidance of long-term complications of Lyme disease, including its attendant costs [21,22].

Changes in clinical trial design also extend to statistical methodology and how to best address missing data. Subjects fail to complete trials for a variety of reasons which may or may not be related to the trial itself. This missing data introduce uncertainty. Biostatisticians consider noncompletion rates ≥20% as excessive because they limit the validity of outcome conclusions [23]. Therefore, studies with a noncompletion rate above this threshold should not be used in therapeutic efficacy determinations. When the noncompletion rate is below the 20% cut-off, missing data can be handled in several ways. The choice of methodology and specific details regarding its application should be established prior to recruitment. Intent-to-treat (ITT) methodology, which is thought to more closely reflect clinical reality, is preferred [24]. Other methodologies, such as complete case (CC) or last value carried forward (LVCF), tend to overestimate treatment effectiveness. In CC analysis, all data are excluded for any subject who does not complete the study. This allows investigators to disregard all who left the trial prematurely [25], including those whose absence was due to either treatment failure or medication intolerance, which are clearly not random events [24]. LVCF methodology assumes that the observed response at the last contact remains constant. Thus, patients who subsequently relapse or experience disease progression after their last visit would be misidentified. 

## 3. US Trial Evidence

A Medline search was conducted on 30 December 2020 to identify prospective, randomized trials conducted in the United States for the treatment of patients with erythema migrans lesions using either amoxicillin, cefuroxime, doxycycline or azithromycin as these were the agents named in the IDSA/AAN/ACR recommendation. The search used this strategy: (erythema migrans OR erythema migrans chronicum) AND (amoxicillin/therapeutic use OR cefuroxime/therapeutic use OR doxycycline/therapeutic use OR azithromycin/therapeutic use) and these filters: clinical trial, randomized controlled trial, English language. There were 25 results; of these, 8 met the search criteria. The other 17 papers were: EM trials conducted in Europe (11), trials of antibiotic prophylaxis of Ixodes tick bites (3), a trial on the treatment of disseminated disease in Europe (1), a European antibiotic re-treatment trial for persistent symptoms of Lyme disease (1) and a paper on the diagnostic utility of culturing skin biopsies from suspected EM lesion (1).

The retrieved US trial evidence is old. As a group, the eight randomized comparative EM trials enrolled patients between 1988 and 1999 [14,15,16,17,26,27,28,29]. In the intervening years, the consensus around what constitutes adequate trial design and execution and treatment success has changed significantly. Of the eight trials [14,15,16,17,26,27,28,29], most investigated the efficacy of amoxicillin, cefuroxime or doxycycline in 20- or 21-day regimens [15,16,26,27,28]. One trial compared 20 days of amoxicillin to 7 days of azithromycin [17]. Another compared three doxycycline regimens of 10- or 20-days duration [29] and the remaining trial used either an initial 10-day regimen of doxycycline or amoxicillin plus probenecid or a 5-day regimen of azithromycin [14]. This last trial was designed such that treatment could be immediately repeated if the investigators judged that therapy had failed.

The US trials were small. Across the eight trials, the largest arm contained 124 subjects while the smallest had 13 [17,28].

Two of the eight trials had noncompletion rates in excess of 40% [16,29] and two others had a single arm with non-completion rates ≥20% [15,28]. Therefore, the results from these trials/arms should not be used to determine treatment efficacy.

Of the six remaining trials, several used disease-centered endpoints that allowed patients with persisting symptoms, and in some cases, objective signs, to be categorized as having satisfactory or even successful outcomes [15,17,27]. In the study by Nadelman et al., treatment “success” was defined as no signs or symptoms of late disease while “improvement” allowed for ongoing signs or symptoms provided there was no “objective evidence of active disease” [15]. Outcome definitions in the Luft et al. trial allowed patients with persistent symptoms to be categorized as “complete response” provided there were no objective signs and at least 75% of the symptoms had cleared [17]. The definition of partial response was especially complicated and allowed for the presence of both signs and symptoms. In the 1997 study by Dattwyler et al., treatment success required only the resolution of objective findings [27].

All six trials used what is currently thought of as outdated statistical methodology—complete case (CC) or last value carried forward (LVCF) [14,15,17,26,27,28]. As previously discussed, these methodologies are prone to overstating treatment success. In the case of Lyme disease, relapse and disease progression following the use of prevailing antibiotic regimens is known to occur [14,17,30]; therefore, LVCF is not an acceptable way to account for missing subjects in Lyme disease treatment trials.

The combined effect of utilizing disease-centered endpoints and inappropriate statistical methodology makes it quite likely that the actual treatment efficacy for achieving patient-centered therapeutic goals, while unknown, is lower than the 85–95% rates that were originally reported by investigators.

## 4. Shortcomings of the IDSA/AAN/ACR Recommendations for Management of Patients with Erythema Migrans Rashes

### 4.1. Inappropriate Reliance on European Data

Although the related text includes more than 30 references, the evidence tables used to establish preferred agents include only six US trials [6]; three of the eight tables, 1, 3 and 6 (in order of appearance), are based strictly on European data. The evidence tables establishing duration of therapy included only two US trials and three of the five tables, 2, 3 and 4 (in order of appearance), are based strictly on European data. As discussed in the background section, there are substantive differences between Bbss and *B. afzelii* and it has not been demonstrated that efficacy findings from European trials are generalizable to US patients. Therefore, the IDSA/AAN/ACR treatment recommendations should err on the side of caution and not be based in whole or in part on European trials [10].

### 4.2. Insufficient US Data Regarding Duration of Therapy 

The IDSA/AAN/ACR treatment recommendation for US patients with EM rashes advises clinicians to prescribe either 10 days of doxycycline or 14 days of either amoxicillin or cefuroxime axetil [6]. If using azithromycin, a second-line agent, the recommended duration is 7 days [6]. However, there are insufficient and, in some cases, no US trial data to support these recommendations with regard to the duration of therapy. There are no US trial data to support 14-day regimens of amoxicillin or cefuroxime as monotherapy. US trials that investigated the use of these antibiotics as monotherapy used 20-day regimens [14,15,16,17,28]. Amoxicillin plus probenecid was used in two US trials [14,26]; one used a 21-day regimen [26] while the other employed 10 days of therapy [14]. The duration evidence tables did include one US trial by Wormser et al. that evaluated a 10-day duration of doxycycline in US patients [6]. Of the 61 patients randomized to this arm, 30 (49%) failed to complete the trial. Although it was not included in the duration evidence tables, another US trial also examined a 10-day doxycycline regimen [14]. Of the 22 subjects in that arm, 7 were immediately re-treated with an additional 10 days of oral antibiotics and an eighth subject experienced disease progression and received ceftriaxone. Thus, in this trial, the clinical failure rate for the 10-day doxycycline regimen was 36%. 

### 4.3. Lack of Patient-Centered Outcomes 

The evidence assessment tables demonstrate that the guidelines authors did not consider critical patient-centered outcomes such as (1) return to pre-Lyme health status, (2) prevention of persistent manifestations of Lyme disease, (3) quality of life improvements (on any validated measure), (4) prevention of EM relapse, (5) and reduction of EM-associated symptoms in their evaluation of the trials [6]. 

This is a critical shortcoming. Persistent symptoms of Lyme disease following antibiotic therapy are common, frequently reported to range between 10–20% [31]. However, the actual incidence is unknown and may be higher [32]; a 2013 observational study of EM patients treated with 21 days of doxycycline found that 33% had ongoing symptoms at the 6-month endpoint [33]. Persistent symptoms can have a profound effect on quality of life and produce functional impairments [4,5,12]. While commonly observed persistent symptoms of Lyme disease are often reported in the non-Lyme population, research using a formalized, research definition of post-treatment Lyme disease syndrome (PTLDS) that included a functional impairment criterion, found that the frequency and severity of such symptoms were substantially different between PTLDS patients and healthy controls [5]. Two examples of these differences: (1) fatigue, present in roughly 60% of controls (none severe) compared to 100% in the PTLDS group (severe in 50%) and (2) difficulty focusing/concentrating, reported by 25% of controls (all mild) compared to 90% in the PTLDS group, (severe in 20%; moderate in 35%).

### 4.4. Low-Quality Evidence 

The most critical outcome that was evaluated in the evidence tables was “Patients experiencing objective findings of Lyme disease (at 6 months and beyond)” [6]. While this is an inadequate surrogate for quality of life, it is useful to look at the evidence strength ratings for this outcome within the seven assessment tables that included US trials. Although the cefuroxime vs. amoxicillin and the azithromycin vs. amoxicillin tables rated the evidence strength as moderate on this outcome, the evidence in four of the remaining trials was rated as low quality. Because subjects in the amoxicillin arm of the trial by Massarotti et al. also received probenecid [14], the findings from this study may not be generalizable to patients receiving amoxicillin alone. Thus, the “moderate” evidence rating in the azithromycin vs. amoxicillin table may be inflated. More importantly, the evidence table evaluating the sole trial that compared 10 vs. 20 days of doxycycline failed to include the 6-month objective findings outcome and instead focused on adverse events. Two other GRADE-based assessments of the trial evidence generally found the evidence to be of low or very low quality [21,22].

In sum, the evidence supporting the preferred agents and duration of therapy in the IDSA/AAN/ACR recommendations is limited in both quantity and quality and fails to consider the outcomes that matter most to patients.

## 5. Evidence-Based, Patient-Centered Therapy

### 5.1. Initial Therapeutic Choices in Individuals at Low Risk for Long-Term Treatment Failure

Patients with a solitary EM rash should receive 20 days of amoxicillin, cefuroxime or doxycycline. In the absence of contraindications, doxycycline is the preferred agent because it is also active against other blacklegged tick-transmitted pathogens such as *A*. *phagocytophilum* [34], *B. miyamotoi* [34], *E. eauclairensis* [34] and *B. mayonii* [35]. 

The evidence supporting the duration of therapy is drawn from the following reanalysis of the US trials/arms of trials discussed above that did not have excessive noncompletion rates [14,15,17,26,27,28]. Reanalyses of previously published randomized controlled trials, based on different statistical approaches and outcome definitions, are not unheard of [36]. To better understand the clinical efficacy of amoxicillin, azithromycin, cefuroxime and doxycycline, from a patient-centered perspective, the original evidence from the six EM trials was carefully reconsidered using current trial design parameters and subsequently grouping findings by agent rather than by trial. Specifically, this reanalysis utilized outcome definitions that reflected patient-oriented goals—restoration of health and prevention of subsequent health problems arising from the initial infection. Thus, treatment success was defined as the complete resolution of the EM rash and all associated symptoms with a return to the pre-Lyme disease health status without subsequent disease relapse or the development of new manifestations consistent with Lyme disease during the observation period. Treatment failure was defined as the occurrence of any of the following: persistent signs and/or symptoms, disease progression or relapse, the emergence of new signs and symptoms consistent with Lyme disease during the observation period not due to re-infections and the premature discontinuance of therapy due to intolerance. When the outcome definitions of an original study differed from these patient-centered definitions, the corresponding original outcome results were re-categorized in accordance with these definitions. For example, subjects originally categorized as “improved” on the basis of reduced but persistent symptoms or findings would be “failures” in this reanalysis.

With regard to the missing longitudinal data, this reanalysis used intent-to-treat (ITT) methodology and choices regarding imputed data reflect the goal of not overstating therapeutic efficacy. Thus, subjects who were excluded from analysis in the original trials due to intolerance of the study drug, loss to follow-up or being designated as “unevaluable” were categorized as treatment failures. 

Table 1 recategorizes the outcome results from the six trials based on patient-centered outcome definitions and the ITT methodology described above. Success rates for two trials are quite uncertain [17,27]. In the Dattwyler et al. paper, 10 subjects reportedly had symptoms at their last visit, which may have not been at the final 9-month visit [27]. Of these 10, it appears that at least 5 would have been assessed at the 9-month endpoint and it is possible that all 10 were. In the Luft et al. trial, patients assessed as complete or partial response at day 20 were subsequently evaluated only for disease relapse and not whether the symptoms and signs present on day 20 had resolved [17]. Although symptom scores were recorded at the 30, 90 and 180-day evaluations, these results were not reported. As such, potential patient-centered success rates for the trial range from 18–81%.

While the success rates for treating EM patients with 20 days of either amoxicillin or cefuroxime and 21 days of doxycycline are lower in this reanalysis than originally reported, and in some instances significantly lower, the findings align with those of a 2013 observational study regarding the treatment of patients with EM rashes [37]. That study demonstrated that 21 days of doxycycline failed to return 39% to their pre-Lyme baseline at 6 months post-treatment, including 11% who had ongoing symptoms and functional decline and 25% with ongoing symptoms alone [37].

Although 20-day regimens of amoxicillin or cefuroxime may perform comparably to a 20-day doxycycline regimen for the treatment of EM rashes in the US, it should not be assumed that shorter courses of these two agents would prove to be efficacious, especially in light of the poor performance by the 10-day doxycycline regimen [14].

Limitations of this reanalysis are: (1) It does not allow for firm conclusions regarding the relative effectiveness of the different regimens. This is due to the low number of subjects for each regimen and the significant uncertainty regarding the outcomes in two of the six trials. (2) It cannot account for the potential impact on efficacy findings from the varied lengths of the observation periods as longer observation periods are more likely to identify disease progression or relapse. Although both cefuroxime trials had a 12-month observation period [15,28], the observation periods in the doxycycline trials were 6–9 months [14,26,27] and the amoxicillin–azithromycin trial had a 6-month observation period [17]. 

### 5.2. Initial Therapeutic Choices in Individuals at Higher Risk of Long-Term Treatment Failure

Clinicians should consider extending the duration of therapy for patients who present with multiple EM lesions, neurologic symptoms or severe symptoms as investigators found such subjects were at increased risk of long-term treatment failure [14,15,16,26]. Long-term treatment failure was also increased in subjects who were ill at the completion of therapy [15,17]. Thus, follow-up contact is necessary to either verify the resolution of all signs and symptoms or to detect ongoing manifestations.

### 5.3. Antibiotic Re-Treatment for Treatment Failures in the Immediate/Recent Post-Treatment Period

Investigators in seven of the eight US trials successfully re-treated some subjects who remained ill or relapsed [14,15,16,17,27,28,29]. Therefore, clinicians should strongly consider doing the same. In order to identify patients who might benefit from re-treatment, clinicians should follow up with all patients at the end of treatment so as to either (1) verify that their symptoms have completely resolved or (2) reassess the need for re-treatment in patients who remain symptomatic, including those whose symptoms are mild. Additionally, clinicians should strongly encourage patients to report any symptoms or signs consistent with disease relapse that occur in the first several months following antibiotic therapy. It is important for clinicians to recognize that the erythema migrans subjects in the original trials who were re-treated represent a distinctly different cohort from those in the NIH-sponsored antibiotic re-treatment trials [38,39,40]; therefore, clinicians are not bound by the reported conclusions of those studies. 

## 6. Conclusions

Lyme disease is an increasing public health threat in the United States. Effective management of patients with erythema migrans is critical to reducing the overall burden of disease on individuals and society. This reanalysis demonstrates that the IDSA/AAN/ACR treatment recommendations regarding EM rashes fail to meet patient-centered therapeutic goals of restoring health and preventing long-term sequalae.

This reanalysis documents the rationale for basing management decisions on case-specific details as different circumstances appear to carry varying degrees of risk. While the limited data suggest that regimens using 20 days of amoxicillin, cefuroxime or doxycycline will be successful in most cases, clinicians should be mindful that a substantial number of patients will fail to return to their pre-Lyme disease health status; the potential for treatment failure may be even greater in certain patient subsets. Patients should be re-evaluated at the end of active treatment and on an “as needed” basis thereafter. In accordance with the actions of some of the US investigators, clinicians should be prepared to offer additional antibiotics if symptoms of Lyme disease persist, recur, or develop in patients with a recent history of an EM rash. Given the scientific uncertainty regarding therapeutic efficacy, the risks and benefits of all options should be discussed with patients in the setting of shared decision-making in order to arrive at a therapeutic plan that fits both the clinical circumstances and the patient’s goals and values. 

This reanalysis also highlights the need for additional research. The mechanisms leading to persistent manifestations have yet to be fully elucidated but several of the trials analyzed in this paper found associations between specific presentations and/or treatment response and persistent manifestations. The optimum duration of therapy, particularly with regard to patient-centered outcomes, has not been established for patients at low risk for long-term treatment failure. Comparative trials using 10-, 20- and 28-day regimens could resolve this important treatment uncertainty. Future research is needed to identify subsets of patients with EM rashes who are at higher risk of treatment failure and to identify initial antibiotic regimens with a higher likelihood of success in those patients. 

## Figures and Tables

**Table 1:** A patient-centered reanalysis of treatment outcomes, by antibiotic regimen, in the randomized comparative erythema migrans trials conducted in the United States.

Author	N	Success	Excluded or Lost to f/Up	Clinical Failure/Re-Treated	Partial Improvement (Reported)	Total Failures
Amoxicillin 20 days						
Luft	122	0–99	19	4	Unclear	23–122
Eppes	13	12	1	0	0	1
**Total**	**135**	**12–111** **(9–82%)**	**20**	**4**	**unclear**	**24–123** **(18–91%)**
Cefuroxime 20 days						
Nadelman	63	34	11	9	9	29
Eppes	15	14	0	1	0	1
**Total**	**78**	**48** **(62%)**	**11**	**10**	**9**	**30** **(38%)**
Doxycycline 21 days						
Dattwyler 1990	37	35	2	0	0	2
Dattwyler 1997	72	48–53	13	1	5–10	19–24
**Total**	**109**	**83–88** **(76–81%)**	**15**	**1**	**5–10**	**21–26** **(19–24%)**
Doxycycline 10 days						
Massarotti	22	14 (64%)	0	8	0	8 (36%)

## Data Availability

The data is available in this manuscript.
